# Severe Lactic Acidosis and Hypoglycemia Associated With Burkitt Lymphoma and the Warburg Effect

**DOI:** 10.7759/cureus.60985

**Published:** 2024-05-24

**Authors:** Pradeep Khanal, Ashbita Pokharel, Sanjog Bastola

**Affiliations:** 1 Internal Medicine, Trinity Health Livonia Hospital, Livonia, USA; 2 Pathology, Corewell Health William Beaumont University Hospital, Royal Oak, USA; 3 Hospital Medicine, Knight Cancer Institute, Oregon Health and Science University, Beaverton, USA

**Keywords:** severe hypoglycemia, lymphoma, lactic acidosis, warburg effect, burkitt lymphoma

## Abstract

Type B lactic acidosis secondary to the Warburg effect is a rare metabolic complication associated with hematological malignancies. Type B lactic acidosis occurs without tissue dysoxia due to increased aerobic glycolysis and excess lactic acid formation, commonly known as the Warburg effect. Here, we present a case of Burkitt lymphoma in a 69-year-old female with severe type B lactic acidosis and hypoglycemia that was effectively treated by the prompt initiation of chemotherapy. Type B lactic acidosis has been mostly described with hematological malignancies and rarely with solid malignancies. It is considered one of the oncological emergencies, and initiation of chemotherapy as soon as possible has been beneficial compared to alkali therapy. Lactic acidosis associated with malignancies carries a poor prognosis and high mortality.

## Introduction

Lactic acidosis (LA) is defined as a whole blood lactate level greater than 5-6 mmol/L and a pH less than or equal to 7.35 [1]. Lactic acid is the end product of the glycolytic pathway under anaerobic conditions. Type A LA is secondary to hypoperfusion and hypoxia and is commonly seen in shock, regional ischemia, and seizures, whereas type B LA is secondary to increased glucose metabolism, which exceeds the oxidative capacity of mitochondria, leading to excess lactate production through the lactic acid cycle. Type B LA is commonly seen in liver disease, human immunodeficiency virus (HIV), thiamine deficiency, total parenteral nutrition, trauma, diabetic ketoacidosis, ethanol intoxication, medications (metformin, epinephrine), and malignancy [2]. LA and hypoglycemia in the latter are due to the Warburg effect. Type D LA is very uncommon and is seen in short gut syndrome and propylene glycol ingestion, thought to be due to intestinal bacterial overgrowth and excessive carbohydrate delivery to the small bowel, leading to the production and absorption of D-lactate [3].

Increased aerobic glycolysis and impaired oxidative phosphorylation by cancer cells are known as the Warburg effect. In tumors, the Warburg effect results from metabolic reprogramming due to the interaction between overexpression of hypoxia-inducible factor (HIF), activation of oncogenes (c-Myc, RAS), loss of function of tumor suppressor genes, activation or inhibition of signaling pathways, and HIF-1 cooperation with epigenetic mechanism [4]. Here, we report a case of Burkitt lymphoma with persistent LA and hypoglycemia in the absence of tissue hypoperfusion or hypoxemia.

## Case presentation

A 69-year-old female with a past medical history of hypothyroidism presented with fatigue and generalized weakness for one month. She also reported a weight loss of 50 lbs in the past one month, along with night sweats, back pain, and progressive oliguria. On presentation to the emergency department, her heart rate was 92 beats/minute, her blood pressure was 115/56 mm Hg, her respiratory rate was 20/minute, her temperature was 97.3°F, and she was saturating well in room air. On physical examination, she appeared thinly built, and chest auscultation revealed clear breath sounds bilaterally. Mild bilateral costovertebral angle tenderness was observed along with bilateral lower extremities edema. Relevant laboratory findings are presented in Table 1.

**Table 1:** Laboratory results on admission

Variable	Result	Reference range
White blood cell count	13.9 K/mcL	3.6-11.1 K/mcL
Hemoglobin	10.4 g/dL	11.4-16.0 g/dL
Sodium	147 mmol/L	135-144 mmol/L
Potassium	3.7 mmol/L	3.5-5.3 mmol/ L
Blood urea nitrogen	64 mg/dL	7-25 mg/dL
Creatinine	2.67 mg/dL	0.60-1.20 mg/dL
Blood glucose	132 mg/dL	70-99 mg/dL
Bicarbonate	19 mmol/L	21-31 mmol/L
Anion gap	31 mmol/L	3-11 mmol/L
Lactic acid	>15 mmol/L	1.5-2.0 mmol/L

**Figure 1:**
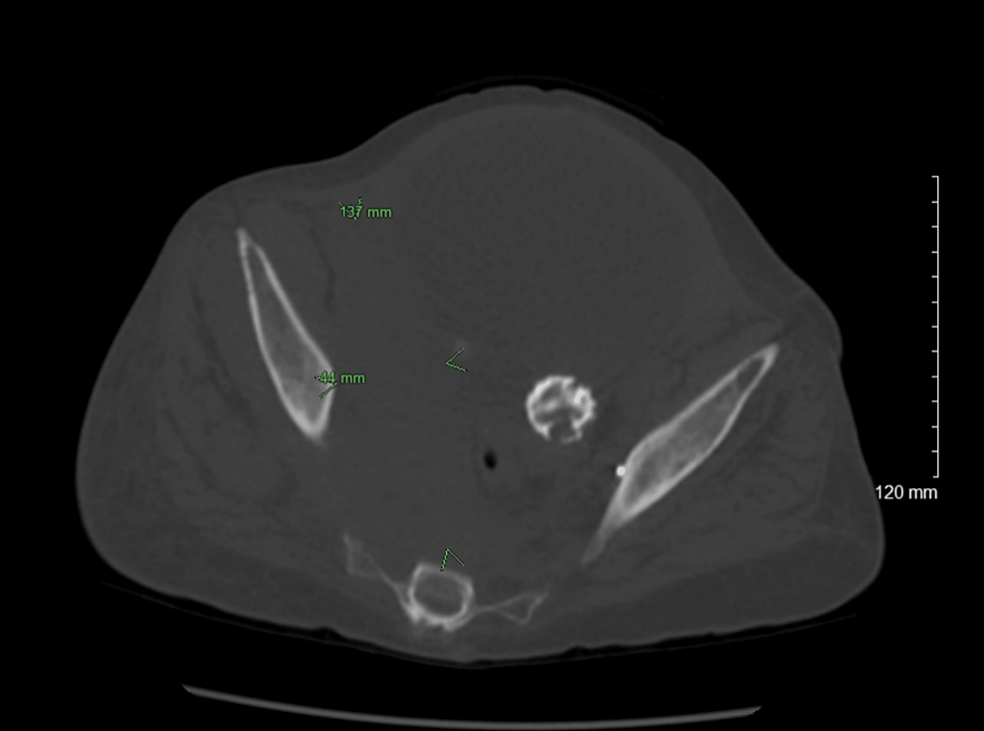
Non-contrast computed tomography of the pelvis demonstrating 4.4×13.7 cm mass (green arrowhead)

**Figure 2:**
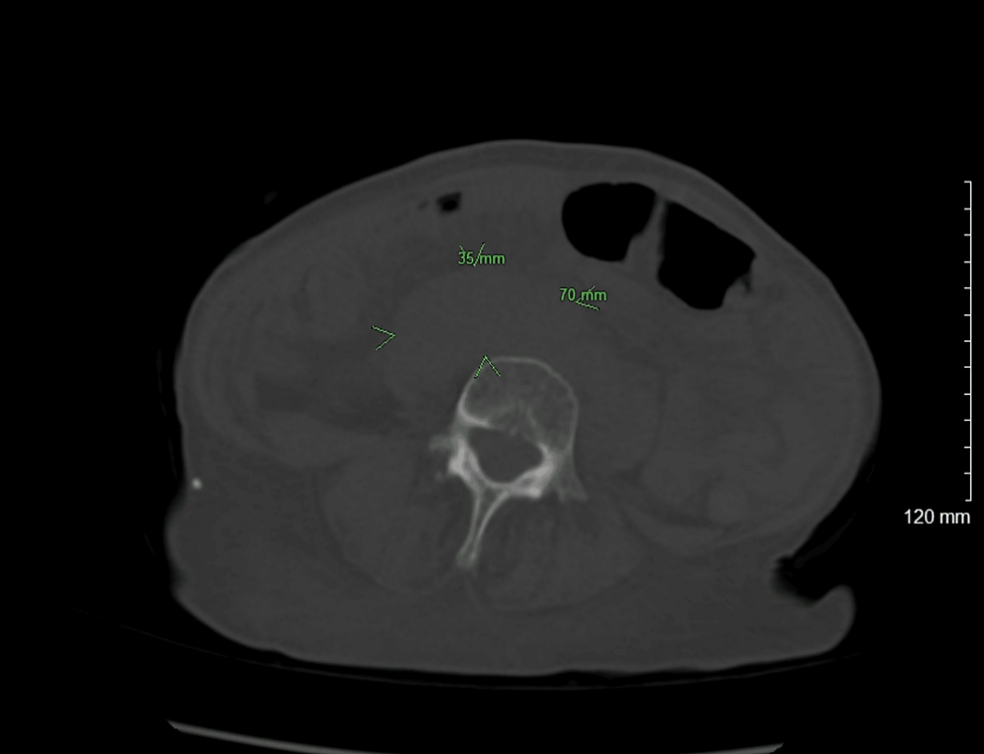
Non-contrast computed tomography of the pelvis demonstrating 7×3.5 cm right retroperitoneal mass (green arrowhead)

Liver function tests and urinalysis were within normal limits. Computed tomography of the chest, abdomen, and pelvis without contrast showed confluent mass-like densities in the retroperitoneum and pelvis, along with probable urinary outlet obstruction and mild bilateral collecting system dilatation. The right pelvic mass measures 4.4×13.7 cm (Figure 1), and the right retroperitoneal mass measures 7×3.5 cm (Figure 2).

**Figure 3:**
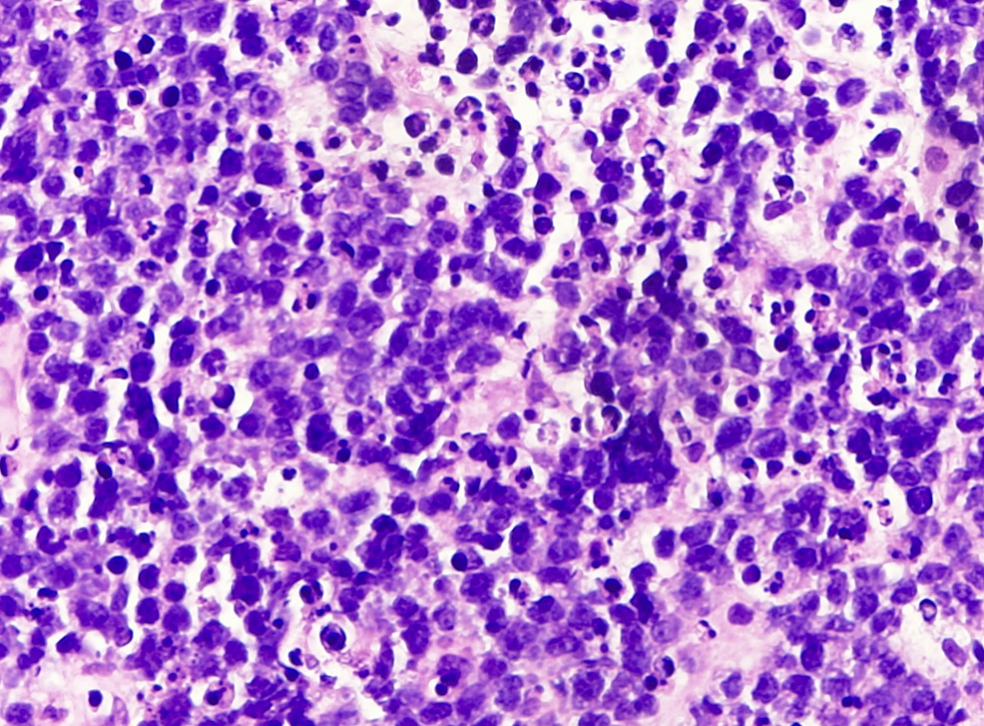
Hematoxylin and eosin stain showing atypical intermediate to large lymphoid cells and apoptotic bodies consistent with Burkitt lymphoma

The patient underwent cystoscopy, and bilateral ureteral stents were placed, which progressively improved creatinine levels. A bilateral lower extremity Doppler revealed acute deep vein thrombosis in the right common femoral, proximal femoral, and gastric veins and in the left gastric vein. She was started on intravenous heparin. Following the administration of 1-liter normal saline bolus, lactic acid improved to 6 mmol/L. A slow infusion of normal saline was continued, but lactic acid slowly started trending upwards. Even though she had a normal glucose level at presentation, blood glucose after 24 hours decreased to 70 mg/dL and continued to be persistently low. Therefore, she was started on 5% dextrose along with sodium bicarbonate drip. Glucose levels eventually improved with continuous dextrose infusion. A biopsy of the right inguinal lymph node was obtained, revealing a diffuse proliferation of intermediate-sized lymphocytes with brisk mitotic activity that are positive for CD20, CD10, BCL-6, and MYC and negative for CD3, BCL-2, MUM-1, TdT, keratin, SOX-10, and synaptophysin. Concurrent flow cytometry demonstrated a monoclonal CD10+ B-cell population. Overall, the findings are consistent with Burkitt lymphoma, as in Figure 3.

**Figure 4:**
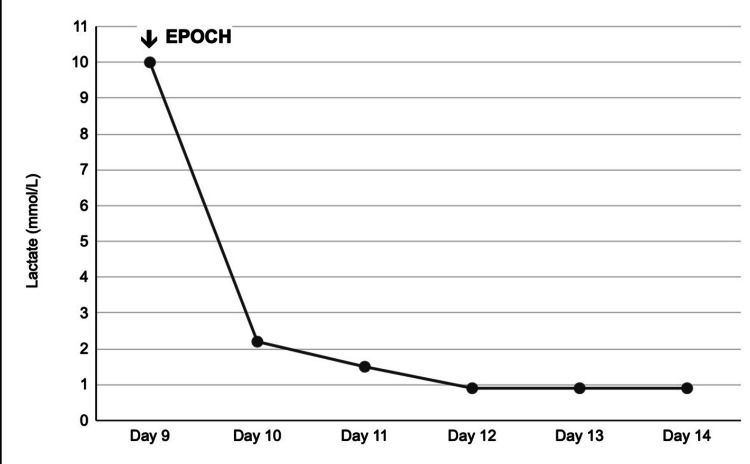
A line graph showing the return of lactate to normal after the start of chemotherapy (EPOCH) EPOCH: etoposide, prednisone, vincristine, cyclophosphamide, and hydroxydaunomycin

The uric acid level was 8.5 mg/dL (reference: 2.3-6.6 mg/dL), and 6 mg rasburicase was given, which decreased the uric acid level to 5.2 mg/dL. She was started on daily allopurinol. On the sixth day of hospitalization, she was started on prednisone 1 mg/kg/day for three days for cytoreduction. On the eighth day of hospitalization, a lumbar puncture was performed, and she was given intrathecal methotrexate. Cytology of the cerebrospinal fluid was negative for malignant cells. On day 9, chemotherapy was initiated with EPOCH (etoposide, prednisone, vincristine, cyclophosphamide, and hydroxydaunomycin), followed by rituximab on day 16 to debulk the tumor. The patient's lactic acid did not improve despite intravenous fluids with a bicarbonate drip and continued to be persistently low. Both lactic acid and bicarbonate levels were normalized within 48 hours of chemotherapy, as shown in the line graph in Figure 4.

The patient was closely monitored inpatient for tumor lysis syndrome and managed accordingly. Later during the hospitalization, she developed altered mental status, neutropenic fever, and sepsis. Ultimately, after discussing with family, the patient was discharged home on hospice care on day 26 of hospitalization.

## Discussion

Lactic acid can be elevated due to either increased production, decreased absorption, or a combination of both. Lactic acid is primarily produced by the muscle, intestine, brain, red blood cells, and skin, whereas it is metabolized by the liver (60%), kidney (30%), and other organs [5]. LA in cancer patients can develop due to both type A (hypoxia) and type B (Warburg effect). Our patient was hemodynamically stable and not on medications associated with LA; however, LA was uptrending, and hypoglycemia was persistent, pointing towards type B due to the Warburg effect.

In 1923, Warburg observed that cancer cells, due to accelerated glycolysis, lead to elevated LA. The acidic environment is suitable for cancer cells to thrive and metastasize while harming the host cell. Lactate not only serves as an oxidative fuel but also plays a role as a potent signaling fuel necessary for the major steps in carcinogenesis, including angiogenesis, cell migration, metastasis, immune escape, and self-sufficiency of cancer cells [6]. The significance of the Warburg effect is utilized in diagnostic testing using fluorodeoxyglucose positron emission tomography [7]. 

Burkitt lymphoma is an aggressive non-Hodgkin's B-cell lymphoma associated with the Epstein-Barr virus, HIV, and chromosomal translocation leading to the overexpression of the oncogene c-Myc. Though the exact mechanism of the Warburg effect in Burkitt lymphoma is not fully understood, it is believed that Myc activates the transcription of genes that encode glucose transporter, hexokinase, pyruvate kinase, pyruvate dehydrogenase kinase, and lactate dehydrogenase, resulting in accelerated aerobic glycolysis, thus Warburg effect [5]. Increased glycolysis in the Warburg effect explains the coexistence of hypoglycemia. Other causes of hypoglycemia in hematological malignancy include increased glucose consumption by tumor cells, infiltration of the liver by tumor, production of an insulin-like substance by malignant cells, and insulin autoimmune syndrome [8]. Despite persistent hypoglycemia, the lack of neuroglycopenic symptoms, as observed in our patient, is supported by lactate serving as a fuel for the brain [9]. 

Type B LA, when associated with malignancy, carries a poor prognosis with a mortality rate of around 90% [10]. Though the best treatment for the Warburg effect in hematological malignancy is not yet known, commonly used modalities for treatment include chemotherapy, bicarbonate infusion, renal replacement therapy, and insulin infusion. Thiamine supplementation has been controversial, as different studies have suggested an anti-tumor effect, while some studies have shown that thiamine supplementation can contribute to a high rate of tumor cell survival, proliferation, and chemotherapy resistance [11]. Among all, initiating aggressive chemotherapy has been effective in the reversal of acidosis [12], as seen in our case as well.

## Conclusions

LA of any type, with or without associated hypoxia, has been associated with a worse prognosis. High anion gap LA without hypoxia in patients with malignancy should prompt the clinician to consider the possibility of type B LA through the Warburg effect. Type B LA is an oncological emergency due to its association with high mortality. Timely identification and initiation of effective chemotherapy have proven to be the most effective methods of reversing acidosis.

## References

[1] Luft D, Deichsel G, Schmülling RM, Stein W, Eggstein M (1983). Definition of clinically relevant lactic acidosis in patients with internal diseases. Am J Clin Pathol.

[2] Foucher CD, Tubben RE (2024). Lactic acidosis. StatPearls [Internet].

[3] Petersen C (2005). D-lactic acidosis. Nutr Clin Pract.

[4] Vaupel P, Multhoff G (2021). Revisiting the Warburg effect: historical dogma versus current understanding. J Physiol.

[5] Looyens C, Giraud R, Neto Silva I, Bendjelid K (2021). Burkitt lymphoma and lactic acidosis: a case report and review of the literature. Physiol Rep.

[6] San-Millán I, Brooks GA (2017). Reexamining cancer metabolism: lactate production for carcinogenesis could be the purpose and explanation of the Warburg effect. Carcinogenesis.

[7] Potter M, Newport E, Morten KJ (2016). The Warburg effect: 80 years on. Biochem Soc Trans.

[8] Ziegler C, Volkov L, Marnai R (2021). Lactic acidosis and hypoglycemia as markers of disease progression of multiple myeloma: a case report. EJHaem.

[9] Elhomsy GC, Eranki V, Albert SG, Fesler MJ, Parker SM, Michael AG, Griffing GT (2012). "Hyper-warburgism," a cause of asymptomatic hypoglycemia with lactic acidosis in a patient with non-Hodgkin's lymphoma. J Clin Endocrinol Metab.

[10] Nair R, Shah U (2017). Lactic acidosis: a rare oncological emergency in solid tumors at presentation. Am J Med Sci.

[11] Lu'o'ng KV, Nguyễn LT (2013). The role of thiamine in cancer: possible genetic and cellular signaling mechanisms. Cancer Genomics Proteomics.

[12] Chan FH, Carl D, Lyckholm LJ (2009). Severe lactic acidosis in a patient with B-cell lymphoma: a case report and review of the literature. Case Rep Med.

